# An Anuran-Based Biotic-Integrity Index for Prioritizing Wetland Conservation Sites in Rwanda

**DOI:** 10.3390/ani16071104

**Published:** 2026-04-03

**Authors:** Selina Glebsattel, J. Maximilian Dehling

**Affiliations:** Institut für Integrierte Naturwissenschaften, Abteilung Biologie, Universität Koblenz, Universitätsstraße 1, 56070 Koblenz, Germany

**Keywords:** monitoring, evaluation, frogs, amphibians, habitat loss, ecozones, freshwater, national parks

## Abstract

We introduce an index based on the composition of amphibian communities that can be used to assess and monitor over time the biotic integrity of wetlands and to evaluate the priority of these sites for conservation. The Rwanda Anuran-based Biotic-Integrity Index (RABI) integrates three sub-indices, which reflect the conservation priority of species based on their distribution in Rwanda, their conservation status, and their susceptibility to habitat alteration. The functionality of the RABI was tested on 51 wetland sites distributed over the five ecozones of Rwanda. The wetland sites showed a wide range of RABI values, with marked differences between the different ecozones. The RABI reliably identified sites with a high number of threatened, range-restricted, and habitat-sensitive species and sites with high species richness. Although wetlands in agriculturally exploited areas often had high anuran-species numbers, their assemblages contained mostly widespread generalist species, resulting in lower RABI values compared to sites with lower species numbers but with threatened, specialized species. Wetlands within the four Rwandan national parks had particularly high RABI values, confirming that these areas require special protection. We identified five sites with high conservation value outside the national parks that should be considered for future protection.

## Simple Summary

In order to conserve biodiversity, it is crucial to protect sites from being altered by humans. In a densely populated country like Rwanda, it is difficult to establish new conservation areas. Therefore, it is important for decision makers to be informed about the conservation value of prospective conservation areas and to be able to prioritize those of high value. Also, it is important to evaluate and monitor the biotic integrity of existing wetlands over time. We present an index that evaluates the conservation value of wetland sites in Rwanda based on the composition of the local frog assemblages. It takes into account the distribution of the species in Rwanda, their conservation status, and their susceptibility to habitat alteration and thereby also reflects the responsibility of Rwanda for the protection of these species. We tested the RABI among 51 wetland sites distributed over the five ecozones of Rwanda and identified sites with high conservation value. Sites with many range-restricted, threatened, habitat-sensitive species have a higher RABI than sites with widespread, non-threatened generalist species. Those with the highest RABI values were found in the national parks, but we also identified sites outside the national parks which should be considered for future protection.

## 1. Introduction

Accelerating biodiversity loss and elevated extinction rates have been widely interpreted as evidence of an emerging sixth mass extinction [1,2,3,4]. Habitat-use change has been the dominant direct driver of recent biodiversity loss worldwide [5]. Biodiversity is important, as a high number of species supports the resilience of ecosystems when it comes to environmental changes [6]. Alteration of the environment, especially the expansion and intensification of land use, deforestation, and urbanization lead to an environmental fragmentation [7,8,9,10]. Human-induced alterations of ecosystems, on the other hand, may jeopardize the livelihood of people, especially in densely populated countries like Rwanda, since the livelihood of numerous people depend on ecosystem services [11,12]. Wetlands in particular play an important role in the support of livelihood as they contribute immensely to securing food and financial income for households in Rwanda. In Rwanda, the total number of wetlands are 935, covering a total area of 176,337 ha [13]. A total of 27% of the Rwandan wetlands are totally protected, whereas 4% are used without specific conditions and another 69% are used under specific conditions [13]. Especially during the dry season, wetlands are agriculturally exploited [14]. The wetlands of Rwanda are under pressure from human population growth, urbanization, agriculture, and climate change effects [13]. Despite a growing population, subsistence farming is still the predominant form of food production in Rwanda [15]. Primary and mature secondary vegetation is decreasing due to high engagement of private households in agricultural activities, especially in rural areas [12].

To quantify and assess changes in environmental quality it is useful to create multimetric indices [16,17]. Bioindicators are the main tool for biomonitoring plans. A total of 97.5% of all publications on multimetric indices to date are related to aquatic bioindicators [18]. Amphibia are among the key groups contributing to total diversity of wetland and providing important ecosystem services [19,20,21,22]. Using anuran amphibians as bioindicators has several advantages: They can be used to assess both terrestrial and freshwater habitats and, due to their wide geographical range, in almost all regions of the world except the polar regions [23]. Furthermore, they react sensitively and quickly to human impact, including habitat destruction, introduced species, and climate [24,25,26]. Almost half of the world’s described amphibian species are declining in population size and 41% are threatened with extinction [27]. Rwanda has a comparatively high diversity of amphibians which is threatened by habitat alteration [26,28]. Few widespread, disturbance-tolerant species benefit from human-altered landscapes, but most of the regionally endemic frog species were found only in the natural habitats [28,29]. Range-restricted and endemic species in particular are often vulnerable to human impact and need special attention in conservation [30]. In order to effectively conserve the remaining diversity of amphibians, and together with them other organism groups in wetlands, it is necessary to identify sites with high conservation priority, i.e., sites that harbour the highest portion of range-restricted, disturbance-intolerant, and threatened species. The aim of our study is to develop an index based on the composition of amphibian assemblages in Rwanda, which can be used to assess, and monitor over time, the biotic integrity of wetland sites, and to evaluate the conservation priority of these sites.

## 2. Materials and Methods

### 2.1. Study Area

Rwanda is a country in the centre of Africa, bordered to the north by Uganda, to the east by Tanzania, to the west by the Democratic Republic of Congo, and to the south by Burundi. It is a small country with only 24,670 km^2^, characterized by rural life and agriculture as one of the most important mainstays [12]. Human population size in Rwanda has tripled in the last half century (1978–2022) and reached 13.2 million inhabitants and a population density of 503 people/km^2^ in 2022 [12]. Rainy seasons are from March to May and from October to December, while dry seasons occur from December to February and June to September [31].

### 2.2. Ecozones of Rwanda

**Figure 1:**
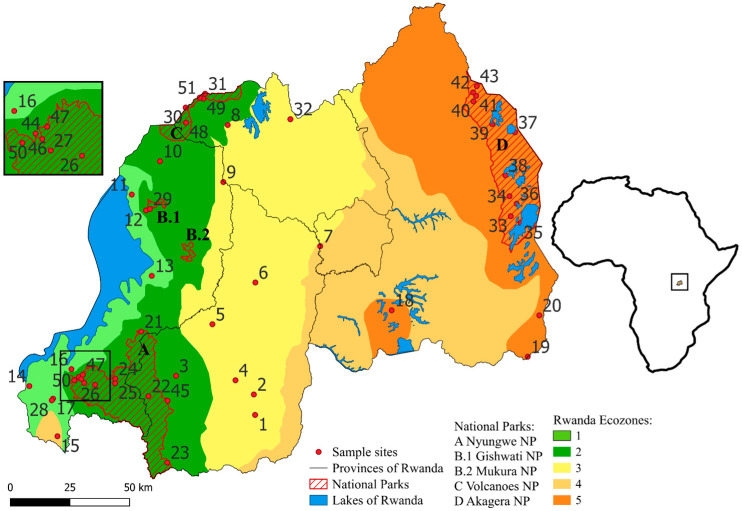
Map of Rwanda showing the 51 sampling localities in the five provinces and the five ecozones. National Parks (NPs): A: Akagera NP, G–M: Gishwati-Mukura NP, N: Nyungwe NP, V: Volcanoes NP. 1: Butare, 2: Karama, 3: Rukarara, 4: Mwogo I, 5: Mwogo II, 6: Gitarama, 7: Kigali, 8: Ruhengeri, 9: Nyabarongo River, 10: Inselberg, 11: Gisenyi, 12: Gishwati–Marais, 13: Kibuye, 14: Cyangugu, 15: Bugarama, 16: Roadside Marais, 17: Cyamudongo–Marais, 18: Mugesera (Gashora), 19: Rusumo, 20: Akagera River, 21: Nyungwe–Source of Nile, 22: Nyungwe–Uwasenkoko, 23: Nyungwe–Nshili, 24: Nyungwe–Rukuzi, 25: Nyungwe–Busoro, 26: Nyungwe–Kamiranzovu, 27: Nyungwe–Karamba, 28: Cyamudongo–Forest, 29: Gishwati–Forest, 30: Volcanoes–Lac Ngezi, 31: Volcanoes–Gahinga Saddle, 32: Rugezi, 33: Muyumbu I, 34: Rwisirabo Hill Pond, 35: Lake Shakani, 36: Lake Birengero, 37: Lake Mihindi, 38: Bweya Ponds, 39: Lake Rwanyakazinga, 40: Kilala Plains I, 41: Kilala Plains II, 42: North of Kilala, 43: Akagera Plains, 44: Gisakura–Marais, 45: Kitabi, 46: Nyungwe–Stream, 47: Nyungwe–Waterfall trail, 48: Volcanoes–Sandi, 49: Volcanoes–Kabatwa, 50: Nyungwe–Ndambarare Waterfall, and 51: Volcanoes–Malalo. Graphic created with QGIS version 3.34 Prizren [35].

We divided Rwanda into different ecological zones (ecozones) which are expected to differ in species composition. Ecozones in Rwanda were previously defined by [11], but their division into six zones was based on elevation and largely coincides with the country’s provincial borders rather than representing boundaries between ecologically relevant parameters. Our approach takes into account data on temperature distribution, rainfall distribution, and soil conditions which were previously used to define agro-climatic zones in Rwanda [32,33,34]. Each of our five ecozones roughly corresponds to 2–3 of the 12 agro-ecological zones of Rwanda defined by [32,34] or 1–3 of the 10 agro-climatic zones defined by [33] as well as the general temperature and rainfall distribution in Rwanda [33]. Temperature and rainfall, and in turn the agro-ecological zones and our ecozones, are to a great part affected by elevation [32]. Based on the combination of temperature distribution, rainfall distribution, and agro-climatic zones according to the categorizations of [32,33], we divided Rwanda into the following five ecozones (EZ; Figure 1).

EZ1 is situated along the shore of Lake Kivu at the western border of the country and lies exclusively in the Western Province at altitudes below 1900 m (Figure 1). It roughly corresponds to the “Impara” and “Lake Kivu Border” agro-ecological zones [32,33,34]. The average annual temperature is 18–20 °C at lower altitudes and around 15–18 °C at higher altitudes. Precipitation is about 1100–1200 mm per year along Lake Kivu and between 1200 mm and >1600 mm in the southwestern Impara region.

EZ2 stretches from north to east, adjacent to EZ1, and comprises parts of the Western, Northern, and Southern Provinces at altitudes between 1900 m and 2500 m (Figure 1). It roughly corresponds to the “Congo-Nile watershed Divide” and the “Birunga” or “Volcanic Land” agro-ecological zones [32,33,34]. All of the remaining montane forest including Volcanoes National Park (NP), Gishwati-Mukura NP, and Nyungwe NP are found within the borders of EZ2. The average annual temperature is 15–18 °C in the lower peripheral areas but below 15 °C at higher altitudes. The higher montane parts in the north and in the south receive more than 1600 mm of rain and the other parts between 1200 mm and 1600 mm.

EZ3 extends from the border with Uganda in the north to the border with Burundi in the south and contains parts of all five provinces but mainly areas in the Northern and Southern Provinces (Figure 1). It roughly corresponds to the “Buberuka Highlands”, “Central Plateau”, and “Granitic dorsal” agro-ecological zones [32,34], the latter being incorporated in the “Central Plateau” by Verdoodt & Van Ranst [33]. It covers much of the Central Plateau at an average altitude of about 1700 m. Altitude in the Buberuka Highlands rises up to 2300 m. The average annual temperature decreases from west to east. The average annual temperature is between 15 °C and 18 °C in the western areas bordering EZ2 and rises to 18–20 °C towards the east. The annual precipitation decreases from about 1200–1600 mm in the western and northern parts to about 1000–1100 m in the eastern parts.

EZ4 contains the eastern parts of the Southern Province, much of the Kigali Province, and the southwestern part of the Southern Province, as well as a small enclave in the southwestern area of the Western Province (Figure 1). It corresponds roughly to the “Eastern Plateau”, “Mayaga” (labelled “Mayaga & Peripheral Bugesera” by [30], and “Imbo” agro-ecological zones [32,33,34]. Altitude is mostly between 1300 m and 1500 m. The average annual temperature is 18–21 °C in most parts of EZ4 but can be over 21 °C in the easternmost and southernmost areas. The annual rainfall is between 900 mm and 1100 mm but less than 900 mm in the north-easternmost area.

EZ5 covers the easternmost parts of Rwanda as well as a small part of the Bugesera region in the southwest of the Eastern Province (Figure 1). It corresponds roughly to the “Eastern Savannas” and “Bugesera” agro-ecological zones [32,34], which were merged to “Eastern Savanna & Central Bugesera” by Verdoodt & Van Ranst [33]. The Akagera NP makes up a large part of the EZ in the east. The landscape is moderately hilly with extensive valleys. The altitude is between 1250 m and 1600 m. EZ5 has the driest and warmest climatic conditions, with an average annual temperature over 21 °C in most parts and annual rainfall of less than 900 mm.

### 2.3. Data Collection

Data on the occurrence of anuran species in Rwanda were taken from [26,36,37], including the taxonomic revision by [38]. We considered a total of 51 individual sites across Rwanda (Figure 1) which had been repeatedly sampled between 2010 and 2023 (for details see [26,28,36]). We calculated a species accumulation curve based on 1000 runs that randomly selected and added the 51 collecting sites.

### 2.4. Rwanda Anuran-Based Biotic-Integrity Index (RABI)

The Rwanda Anuran-based Biotic-Integrity Index (RABI) is inspired by the “Dragonfly Biotic Index” (DBI) [27] which was refined for Rwanda as the “Rwanda Dragonfly Biotic Index” (RDBI) by [11]. It is likewise composed of three sub-indices, (1) the Distribution-based score (DBS), (2) the Threat-based score (TBS), and (3) the Sensitivity-based score (SBS), which are determined for each species to be considered [11,30].

The Distribution-based score (DBS) reflects the conservation priority of a species based on its distribution in Rwanda. It takes into account in how many of the five defined ecozones a species is found and whether it is endemic to Rwanda (Table 1). The distributional restriction of a species to Rwanda means a high responsibility of Rwanda for the conservation of the species. Every species receives at least 1 score point, allowing for prioritization based on the number of species among otherwise similar habitats and for monitoring species richness at a specific site over time.

The Threat-based score (TBS) is based on the categories of the IUCN Red List [11,30,39]. We used the proposals of [26] for the national amphibian red list of Rwanda according to the IUCN red list categories (Table 1).

The Sensitivity-based score (SBS) provides information on how susceptible a species is to changes in its habitat. Anurans that predominantly occur in disturbed areas are apparently less susceptible to effects of human alteration of their environment than the ones which are restricted to natural habitats [30]. The categories of this sub-index were adapted from the information provided by [26] (Table 1).

Our approach differs from the DBI and RDBI [11,30] in the calculation of each of the three sub-indices and in the calculation of the RABI for a specific site. Scores of individual sub-indices range from 1 to 8 (DBS), 0 to 8 (TBS), and 0 to 4 (SBS), depending on different categories for each sub-index (Table 1). Score points double with each successive category, indicating that species in a given category have twice the conservation priority of those in the next lower category. For example, for the DBS, species are assigned 1, 2, 4, or 8 score points. Species such as *Leptopelis bocagii,* which are found in 4–5 ecozones, are assigned one score point. Other species, like *Hyperolius cinnamomeoventris*, are found in only 2–3 ecozones and therefore belong to the next higher category and are assigned 2 score points, reflecting the higher protection priority due to their more limited distribution. Species like *Afrixalus phantasma* that are distributed in a single ecozone are in the next higher category and are assigned yet twice as many score points (i.e., 4) than the species in the previous category. To reflect Rwanda’s high responsibility for the protection of species that are restricted to a single ecozone and are endemic to the country (*Hyperolius jackie*), these species are in the highest category and are again assigned twice as many score points (i.e., 8) than the ones restricted to a single ecozone but not endemic to Rwanda. Thereby, the presence of range-restricted, threatened, habitat-sensitive species will have a stronger impact on the RABI than the species richness of a site. From the sub-indices, the Species Index (SI) is calculated as the sum of the scores of the three sub-indices for each anuran species, ranging from 1 to 20.

SI_species n_ = DBS_species n_ + TBS_species n_ + SBS_species n_

Species with low SI values tend to be widespread, non-threatened habitat generalists, whereas species with an SI closer to 20 tend to be range-restricted, threatened habitat specialists. The RABI for a specific site is calculated as the sum of the SI of all amphibian species recorded at the site.

$$RABI=\sum_{n=1}^{N}SI\;species1+SI\;species\;2+SI\;species\;3+\left[\ldots \right]+SI\;speciesN\;$$

The RABI score is “0” if no anuran species at all is present. Examples for calculating the SI for a species and the RABI for a site are presented in Table 2 and Table 3, respectively.

### 2.5. Data Analysis

We used a species accumulation curve to check the quality of our sample size. We also performed a correlation test to find out how the sub-indices relate to each other. We used Pearson’s chi-squared test to check the differences in species composition within the ecozones. All tests were performed using R (version 4.4.1) [40] with the packages vegan (version 2.6-6) [41], ggplot2 (version 3.5.1) [42], dplyr (version 1.1.4) [43], psych (version 2.4.3) [44], tidyr (version 1.3.1) [45], and tidyverse (version 2.0.0) [46].

## 3. Results

### 3.1. Distribution and Species Richness of the Sample Sites

**Figure 2:**
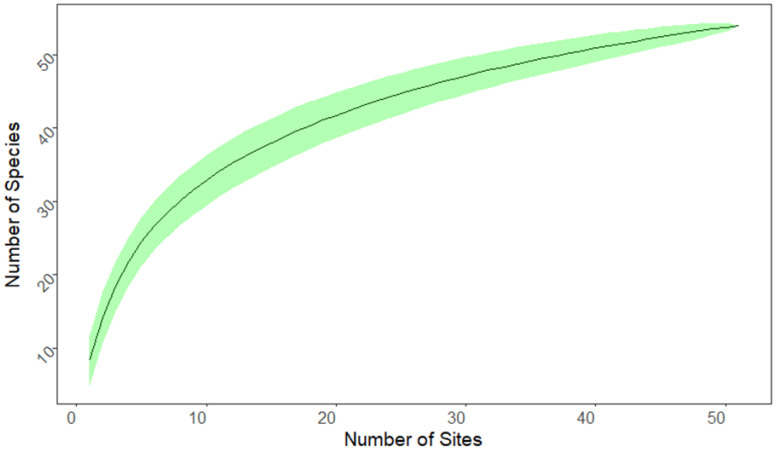
Species accumulation curve for site samples across Rwanda. Green band represents standard deviation based on 1000 runs. The number of species still increases after sampling 51 sites.

The 51 sampling sites were distributed unequally across the five ecozones (Figure 1; Table 4). The minimum of two sampling sites was found in EZ4 and the maximum of 21 sites in EZ2. A total of 54 out of at least 61 known anuran species recorded from Rwanda [26] were documented. The number of species recorded at a single site varied between 2 (site 44) and 17 (site 1) (Table A1). The overall mean species number per site was 8.3 (Table 4). The mean species number per site was much lower in EZ2 (6.2) and EZ5 (7.9) than in EZ1 (10.0), EZ3 (12.3), and EZ4 (12.5) (Table 4). The most frequently recorded anuran species in general were *Hyperolius kivuensis* (N_Sites_ = 24), *Amietia nutti* (N_Sites_ = 22), and *Ptychadena nilotica* (N_Sites_ = 21), whereas about half of the species (N = 25) were detected at only a single site (Table A2). The species accumulation curve had not reached saturation after 51 sample sites, indicating that sampling of additional sites will probably yield additional species (Figure 2).

### 3.2. Species Richness and Species Composition of the Ecozones

The highest accumulated species number (N = 32) was found in EZ2 and the lowest number in EZ4 (N = 18); the other ecozones showed a species number between 22 and 24 (Table 4, Table A1). EZ2 also had the highest number of unique species (N = 13), i.e., species that were recorded only in that particular ecozone within Rwanda. In the other zones, only two (8.3%; EZ1 and EZ3), three (16.7%; EZ4), and five (22.7%; EZ5) species were unique (Table 4). The percentage of unique species in EZ2 (40.6%) was almost twice as high as in EZ5 (22.7%) and much higher than in EZ1, EZ3, and EZ4 (8.3–16.7%; Table 4, Table A3).

A similar pattern was observed in the proportions of species that were shared between the different ecozones (Table 5). Between 21.9% (EZ2 with EZ4) and 77.8% (EZ4 with EZ3 and with EZ5) of the species were shared between two separate ecozones. The proportion of shared species was consistently lower in EZ2 (minimum 21.9% with EZ4, maximum 46.9% with EZ1) than in the other ecozones (Table 5), whereas EZ4 showed the highest rate of shared species with more than 72% of species shared with EZ1, EZ3, and EZ5. EZ1 shared most of the species with EZ2 and EZ3 (both 65.5%), EZ2 with EZ1 (46.9%), EZ3 with EZ5 (66.7%) and with EZ1 (62.5%), EZ4 with EZ3 and EZ5 (77.8%), and EZ5 with EZ3 (72.7%) (Table 5). The results of the Pearson’s chi-squared test indicated differences in the composition of species in the different ecozones (χ-squared = 252.11, df = 212, and *p* < 0.03).

### 3.3. Species Index and Sub-Indices

The values of the sub-indices were not distributed equally among the anuran species (Table 6). Regarding the DBS, about half of the species (N = 25; 46.3%) were recorded in only one ecozone and thus belong to the second-highest DBS category (4 points), whereas only a single species endemic to Rwanda belonged to the highest category (Table 6). A little more than half of the species were in the lowest TBS category and another third in the second-highest (Table 6). About half the species were found in the two lower and the other half in the two upper SBS categories (Table 6).

**Figure A1:**
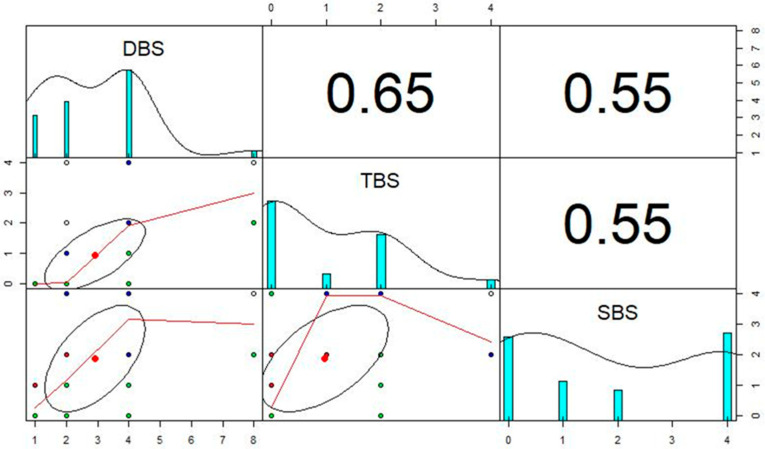
Results of the correlation test, showing moderately strong correlations between the sub-indices. The bar charts on the diagonal show the distribution of the respective sub-indices. Below on the left, scatter plots show individual observations as dots, dispersion/covariances as ellipses, and the regression as a red line. Coefficients of the correlations between respective pairs of sub-indices at the top right indicate moderately strong correlations between the sub-indices.

In correlation tests, all three sub-indices showed moderately strong positive correlations with similar correlation coefficients between the three sub-indices (SBS–TBS, r = 0.55; DBS–TBS, r = 0.65; and DBS–SBS, r = 0.55) (Figure A1).

**Figure 3:**
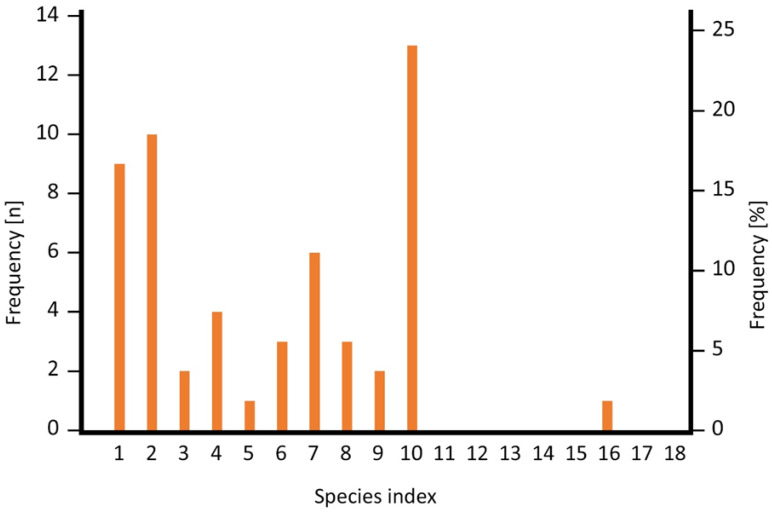
Frequency (left scale) and percentage (right scale) of SI values among the 54 anuran species recorded from 51 wetland sites in Rwanda.

The SI values resulting from the combined sub-indices scores show a wide range between 1 (lowest possible value; n = 9) and 16 (n = 1), the latter having been assigned to *Hyperolius jackie* which is considered endemic to Nyungwe Forest in Rwanda. The highest possible value of 20 was not assigned to any of the species. The species were unevenly distributed among the values (Figure 3), the maximum number of species (N = 13) had an SI of 10, and another peak was at the lower end of the scale at 2 (n = 10) and 1 (n = 9). The SI values are distributed mostly in the lower half of the spectrum of possible values, and the median SI was 6. We present a list of the sub-indices (DBS, SBS, and TBS) and the resulting SI for all 54 anuran species in Table A4. Species in montane forest had an SI from 1 to 16 with a median of 8, whereas species in lowland (farmland and savannah) had an SI from 1 to 10 with a median of 2.

### 3.4. RABI and Sites of High Priority for Conservation

Across all ecozones, the RABI ranged from 5 (site 34, EZ5; sites 44 and 45, EZ2) to 63 (sites 22 and 29, EZ2). In EZ1, the RABI values ranged from 11 to 47 (Table 7). Sites in EZ2 had the widest range of the RABI, spanning the whole range from 5 to 63 (Table 7). 16 of the 21 sites, however, had a RABI > 20, with the exceptionally lower values being restricted to non-forest sites within the ecozone (Table A1). The RABI of sites in EZ3 ranged from 14 to 38 (Table 7), with values above 20 being the exception. The two locations in EZ4 had RABI values of 16 and 29 (Table 7). EZ5 had a wider range of the RABI, with values between 5 and 30 (Table 7).

The median RABI of sites was highest in EZ2 ($\tilde{x}$ = 33), followed by the median site RABI in EZ4 ($\tilde{x}$ = 22.5) (Table 7). They were about twice or one-and-a-half-times higher, respectively, than the median RABI of sites in EZ1 ($\tilde{x}$ = 15.5), EZ3 ($\tilde{x}$ = 18), and EZ5 ($\tilde{x}$ = 14.5) (Table 7).

**Figure 4:**
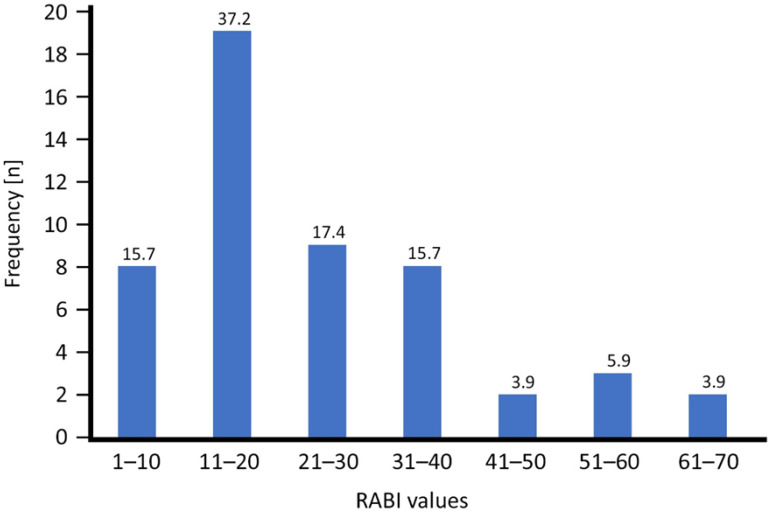
Frequency (total number) of categories of RABI values among 51 wetland sites in Rwanda. Numbers above bars are corresponding percentage values.

**Figure 5:**
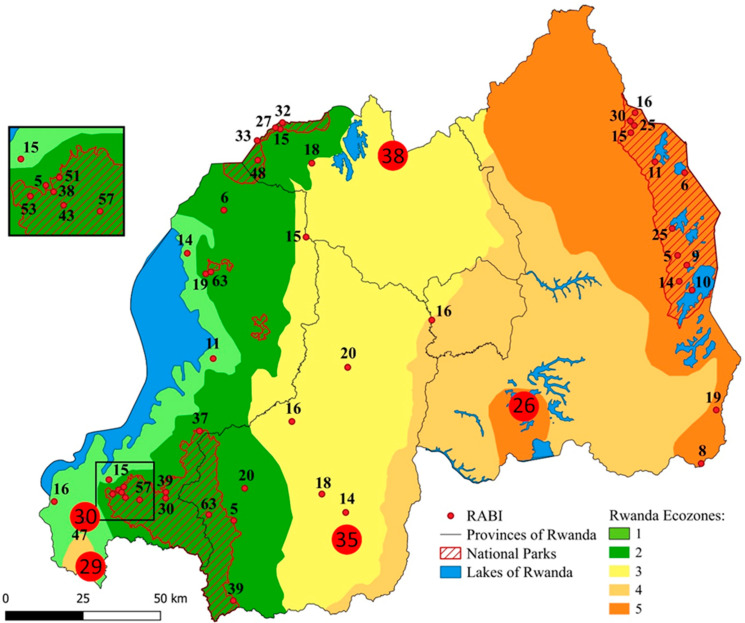
RABI scores of the 51 sampling localities (see Figure 1) in the five ecozones of Rwanda. Most sites of high conservation priority are situated in one of the four national parks. Five high-conservation-priority sites outside the national parks are highlighted as large red circles with their respective RABI score in the centre.

More than half of the wetland sites (N = 27) had a RABI of 20 or less, whereas seven sites (13.8%) had a RABI > 40 (Figure 4). Most of the sites with high conservation priority (RABI > 20) are located inside one of the four national parks, but we identified five sites of high conservation priority outside the national parks among our samples (Figure 5), i.e., the wetlands outside Cyamudongo Forest (site 17, Ecozone 1; RABI 30; 11 spp.), at Butare (site 1, Ecozone 3; RABI 35; 17 spp.), the Rugezi Marshland (site 32, Ecozone 3; RABI 38; 10 spp.), Bugarama (site 15, Ecozone 4; RABI 29, 13 spp.), and Gashora/Mugesera (site 18, Ecozone 5; RABI 26, 15 species).

## 4. Discussion

### 4.1. Distribution and Species Richness of Sample Sites, Composition of Ecozones

The 51 sampling sites considered in our study were not equally distributed across the five ecozones with only two sites in EZ4 and 21 sites in EZ2. EZ4 is underrepresented in our data set, and additional sites in EZ4, and to a lesser extent in EZ1 and EZ3, should be prioritized for future field work in the course of a country-wide assessment of the anuran diversity in Rwanda. Despite the low number of accessed sites, the total number of species in EZ4 (N = 18) is only slightly smaller than in EZ1 (N = 24), EZ3 (N = 24), and EZ5 (N = 22). Additional field work will likely substantially increase the total record of species from that EZ. The highest accumulated overall species number (N = 32) and, simultaneously, the lowest mean species number per site (6.2) were observed in EZ2, confirming the results of previous studies that found the lowest local (alpha) but the highest regional total (gamma) amphibian diversity in Rwanda in the montane forest zone [26,28,36]. The fact that EZ2 has the lowest portion of species shared with other EZs and therefore has the by far highest percentage (40.6%) of species unique to the particular EZ contributes to the generally high RABI scores (median 33) of sites in EZ2. In contrast, the percentage of unique species in the other lower EZ with mostly open vegetation ranged only between 8.3 (EZ1) and 22.7% (EZ5). This is in agreement with the observation that farmland in Rwanda contains highly homogenized amphibian assemblages that have comparatively high species numbers but mainly consist of widespread, disturbance-tolerant, non-threatened species [26,28]. Although the taxonomic alpha diversity (species richness) of amphibians at a given site in altered habitats is higher than in natural habitats, species turnover is much lower, resulting in reduced taxonomic, functional, and phylogenetic gamma diversity in farmland in comparison to natural habitats across Rwanda [28]. Few disturbance-tolerant species benefit from human-altered landscapes, but most of the regionally endemic frog species were found only in the natural habitats [28,29]. Refs. [26,28] emphasize that endemic and functionally specialized amphibian species are restricted to the national parks in Rwanda. Two kind of regions have priorities in conservation: firstly, regions with a high species richness, and secondly, regions with many rare species [47]. As our RABI is both affected by species richness and rarity, it reflects these priorities.

### 4.2. Rwanda Anuran-Based Biotic-Integrity Index (RABI)

Amphibians contribute significantly to the total diversity of wetlands and provide important ecosystem services [19,20,21,22,28,29,48]. Their highly permeable skin makes them prone to take up and accumulate contaminants from water, soil, and even air, making them valuable indicators of environmental pollution across a wide range of habitats [49]. Amphibians are sensitive indicators of bioclimatic stress and environmental degradation and are among the first vertebrates to respond to climatic and environmental fluctuations, making them excellent indicators for ecosystem change [22].

Anurans are particularly well-suited as bioindicators for biological monitoring programs and habitat integrity assessments of wetlands which provide the habitat for both the terrestrial post-metamorphic life stages and the aquatic larvae. The larvae (tadpoles) of most species develop in water and react quickly to changes in the water quality. The terrestrial life stages also depend on the wetland as habitat and, therefore, changes above and below the water surface might affect an anuran species’ presence in the wetland. Amphibians and their larvae make up a large part of the vertebrate biomass in many ecosystems and serve both as prey and as primary and secondary consumers. Due to their relative abundance, they are comparatively easily detected; most species are also relatively easy to identify to species level based on external morphology. In addition, males of most anuran species emit a species-specific call that allows for human observers to identify a calling frog to species level without the need to see or even collect it [50]. For the assessment of species presence and abundance, the bioacoustic approach, i.e., standardized recordings of male anurans advertising in a given microhabitat type complemented with visual species detection and identification, has been successfully applied for diversity assessments in afro-tropical wetlands [26,36,51,52,53,54]. Keys to all Rwandan amphibian species based on external morphology as well as call properties are available [26].

The Rwanda Anura Biotic Index, which we introduce here, is inspired by the “Dragonfly Biotic Index” (DBI) [30] and the “Rwanda Dragonfly Biotic Index” (RDBI) [11] and calculated in a similar manner. Like the predecessors, it is composed of three sub-indices, the DBS, TBS, and the SBS, which are determined for each species to be considered [11,30]. We refer to the sum of the three sub-indices for each species as the “Species Index” (SI) to avoid confusion with the term “RABI” which we only apply to sites. Furthermore, for the calculation of the RABI, we chose to use the sum of the SI of all species present at the site (equal to the “total DBI score” of [30]) but not to divide the sum by the number of species. Thereby, a site with a higher number of species will receive a higher RABI than a site with a lower species number, provided that species properties are otherwise similar. This will not only allow for comparisons between two different sites in their current state but also to reveal changes in the number of species present at a single same site in the course of time during monitoring programs. Even if two or more sites harbour only widespread, non-threatened, non-sensitive species, the RABI will indicate which site has a higher conservation priority and suggest a decision for which one to choose if one had limited means and could protect only one of the sites. The subsequent higher DBS categories are appointed 2, 4, or 8 points. The maximum DBS value for a range-restricted species endemic to Rwanda reflects the high responsibility of Rwanda for the protection of the species. Our approach further differs from the DBI and RDBI in the scalation of the scores of the sub-indices, i.e., exponential, not linear. The appointment of twice as many score points in every higher category corresponds better to the elevated conservation need for respective species than the scoring with a linearly graded scalation, especially in higher categories.

Like the DBI and the RDBI, we use the entire species assemblages at a site to assess the quality and the conservation value of the wetland sites [11,30]. The use of the entire assemblages is more likely to accurately characterize the ecological integrity of a habitat than the use of single indicator taxa with specific habitat [55].

The combination of three sub-indices which are each based on different information about each species present in the community provides a sense of the conservation value [11]. We have defined four categories of each of the three sub-indices (DBS, TBS, and SBS). The results show that the species are well distributed over the four categories. About half the species are found in the two lower categories of the DBS and SBS, respectively, and in the single lowest category of the TBS. The other half of the species are distributed over the two or three higher categories, respectively. This indicates on the one hand that the categories of the sub-indices are well defined to reveal differences between the Rwandan anuran species and on the other hand that the Rwandan anurans are heterogenous enough to fit into different categories and are in general a well-suited group to be assessed through a category-based index.

The distribution of species in different categories of the DBS, TBS, and SBS differs between Rwandan anurans and odonates. With respect to the distribution (DBS sub-index), about half of the anuran species were in the two lowest categories, almost half in the second-highest (restricted to a single ecozone), and only one species (*Hyperolius jackie*) in the highest category of the DBS category. In contrast, about 75% of the Rwandan odonate species are widespread and only about 15% are restricted to one or two ecological zones [11]. A little more than half the Rwandan anuran species are considered to be not threatened (“Least Concern”) and belong to the lowest TBS category; the remaining species are distributed over the other four threat-based categories. In Rwandan odonates, on the other hand, almost 98% belong to the lowest TBS category and 82% in the lower two SBS categories [8]. Therefore, assessments of the two groups in Rwanda are expected to yield somewhat different results, and simultaneous assessments of both groups could well complement each other when evaluating wetlands.

The SI values resulting from the combined sub-indices scores show a wide range between 1 (lowest possible value, n = 9) and 16 (n = 1). The highest possible value of 20 was not assigned to any of the species. Anuran-species assemblages in Rwanda differ between montane forest and savannah and farmland; most species found in farmland naturally occur in open wetlands like savannahs, whereas only very few forest species can cope with farmland [28]. As montane forest is restricted to elevations above approx. 2000 m, the differences in anuran-assemblage composition correlate with altitude in Rwanda [26,28]. Species in montane forest and wetlands are often specialists that are only found in one or a few locations, and—largely because of that—many of them have been classified as threatened [26]. In contrast, the species of the lowlands are mostly non-threatened generalists with a wide distribution in Rwanda and beyond [26,28]. There are, however, some notable exceptions of range-restricted lowland species in Rwanda which are classified as threatened and nowadays restricted to natural savannah in Akagera National Park [26]. Montane species had on average a much higher SI (median 8) than species from farmland and savannah (median 2) which has a great impact on the RABI of the sites.

### 4.3. RABI and Sites of High Priority for Conservation

The degree of homogenization of sites is largely reflected by the RABI. Apart from three notable exceptions, all farmland sites in EZ1–EZ5 had a RABI of 5–20. On the other hand, all sites but one in the three montane forest national parks and three sites in Akagera National Park had a RABI of 25 or higher, resulting from the prevalent presence of threatened, range-restricted, habitat-sensitive species at these sites. Most of the sites with a RABI > 20 are located inside one of the four national parks and are therefore already protected. Of the five wetlands sites with the highest conservation priority outside the national parks, only one already has a protective status, i.e., the Rugezi Marshland (site 32, Ecozone 3; RABI 38; 10 spp.). It harbours rare species like *Afrixalus orophilus*, *Hyperolius lateralis*, *Phrynobatrachus bequaerti*, and *Ptychadena chrysogaster* [26,54], along with threatened bird species [56,57]. Rugezi Marshland encompasses 3% of Rwanda’s total wetland area [10]. Designated in 2005, it remains the only Ramsar [58] site in Rwanda, although 60 additional sites, representing 53% of Rwanda’s total wetland area, are proposed for Ramsar status [13]. Among these prospective Ramsar sites is Mugesera-Rweru which we also identified as a site of high conservation priority (site 18, Ecozone 5; RABI 26, 15 species, including *H. lateralis*). Two other sites (site 17 outside Cyamudongo Forest and site 15 Bugarama) harbour a high number of species with a high proportion of species not found elsewhere in Rwanda such as *Leptopelis* cf. *cynnamomeus* and *Phrynobatrachus* sp. A (as well as the caecilian *Boulengerula fischeri*), and *Ptychadena guibei*, *Hoplobatrachus occipitalis*, and *Xenopus muelleri*, respectively [26]. The fifth site is the agricultural wetland at Butare, the site with the highest number of species (17) so far recorded in Rwanda, including species such as *Ptychadena uzungwensis* and *Hyperolius lateralis* that have been recorded from only a few other locations in the country [26,29,52].

Wetlands are dominant features of the Rwandan landscape, and Rwanda has already taken the significant step of recognizing the importance of wetlands and the need to manage them proactively [13]. The government of Rwanda counts 935 wetlands which cover a total area of 176,337 ha [59]. A total of 27% of the Rwandan wetlands are totally protected, whereas 69% are used under specific conditions and another 4% are used without specific conditions [59]. Of these, approximately 31,000 ha (64% of wetlands under total protection and 17% of all wetlands) are formally protected within a national park [59]. The remaining 17,021 ha (36% of totally protected wetlands) have limited protection in reality and are vulnerable to livestock and cultivation encroachment, and poaching of wildlife [59]. The RABI is a tool that helps to evaluate the value of wetland sites and to make decisions about attributing protective status. The national wetland management framework for Rwanda calls for environmental impact assessments (EIAs) in order to evaluate wetlands with regard to possible qualification as a site of international, national, regional, or local importance [59]. The RABI complements similar indices such as the RDBI as well as assessments of coverage of natural vegetation, soil type, and presence of rare bird species that were used in previous evaluations of wetlands in Rwanda (e.g., [59]).

Although our paper focusses on Rwandan wetlands, the assessment of wetland conservation priorities is of international relevance. The methodological definition of categorical parameters standardizes the assessment of wetlands based on amphibian species assemblages. The RABI (or more generally the “Amphibian-based Biotic-Integrity Index” [ABI]) can be applied to any country or other defined region with amphibian populations. The ABI requires the definition of ecozones, the existence of a national red list, and an inventory of amphibian species, including the quality of the wetlands in which they are found. If the number of defined ecozones differs from those in Rwanda, the categories of the DBS would need to be adjusted accordingly.

## 5. Conclusions

We developed an index based on the composition of amphibian communities that can be used to assess and monitor the biotic integrity of wetlands over time and to evaluate the priority of these sites for conservation. The definitions of the three sub-indices, on which the index is based, have proven to be useful to differentiate the species’ conservation need over a wide range. The RABI allows us to assess the conservation value of a specific site which can be used for prioritization among sites. The wetland sites showed a wide range of RABI values, and there were marked differences in the RABI values between the different ecozones. The RABI was able to reliably identify sites with a high number of threatened, range-restricted, and habitat-sensitive species and sites with a high species richness. Wetlands within the four national parks (especially in the forested and montane regions) had particularly high RABI values, confirming that they require special protection. Although wetlands in agriculturally exploited areas often have high anuran-species numbers, these assemblages show a shift towards widespread generalist species, resulting in lower RABI values compared to sites that have lower species numbers but harbour threatened specialized species. The RABI proved to be useful to evaluate sites in Rwanda, and with few modifications regarding the DBS and the red list status of species in the respective countries or regions, it can be applied to other regions in the world.

## Figures and Tables

**Table 1:** Summarizing description of the three sub-scores SBS, TBS, and DBS in their different categories. The TBS is based on the IUCN Red List categories [39]. LC = Least Concern, NT = Near Threatened, VU = Vulnerable, EN = Endangered, CR = Critically Endangered, and DD = Data Deficient. n/a = not assigned.

Score	DBS	TBS	SBS
0	n/a	LC	wide range of habitats including various microhabitats in disturbed areas
1	present in 4–5 ecozones	NT	few microhabitats in disturbed areas
2	present in 2–3 ecozones	VU/DD	tolerates only near-natural habitats in disturbed areas
4	present in 1 ecozone	EN	only in undisturbed habitats
8	present in 1 ecozone and endemic to Rwanda	CR	n/a

**Table 2:** Exemplary calculation of the Species Index of *Arthroleptis adolfifriederici*.

Species	DBS	TBS	SBS	Species Index
*Arthroleptis adolfifriederici*	2	0	4	2 (DBS) + 0 (TBS) + 4 (SBS) = 6

**Table 3:** Exemplary calculation of the RABI for a specific location, site 10 (Inselberg, number of species: 4; Figure 1).

Site	Species	Species Index	RABI
10 (Inselberg)	*Amietia nutti*	1	SI*_A_. _nutti_* + SI*_H_. _glandicolor_*+ SI*_S_. _gutturalis_*+ SI*_S_. _kisoloensis_*= 1 + 2 + 1 + 2 = 6
	*Hyperolius glandicolor*	2	
	*Sclerophrys gutturalis*	1	
	*Sclerophrys kisoloensis*	2	

**Table 4:** Distribution of the sampled wetland sites across the ecozones. For each ecozone, the total number of species recorded, the mean number of species per site, the number and percentage (in parentheses) of species uniquely recorded, and the most commonly recorded species are given.

EZ	N Sites	Total Number of Species Recorded	Mean Number of Species per Site	Number (and Percentage) of Unique Species	Most Commonly Observed Species
1	6	24	10.0	2 (8.3%)	*Sclerophrys kisoloensis*
2	21	32	6.2	13 (40.6%)	*Hyperolius castaneus*
3	8	24	12.3	2 (8.3%)	*Amietia nutti, Hyperolius kivuensis*
4	2	18	12.5	3 (16.7%)	*Amietia nutti, Ptychadena anchietae, Phrynobatrachus kakamikro, Hyperolius rwandae, H. kivuensis, Afrixalus quadrivittatus*
5	14	22	7.9	5 (22.7%)	*Ptychadena nilotica, Kassina senegalensis*
total	51	55	8.3	n/a	*Hyperolius kivuensis*, *Amietia nutti*, *Ptychadena nilotica*

**Table 5:** Number and percentage (in parentheses) of anuran species shared between the five ecozones of Rwanda.

Ecozone	Total Number of Species Recorded	Shared with EZ1	Shared with EZ2	Shared with EZ3	Shared with EZ4	Shared with EZ5
1	24	24 (100%)	15 (62.5%)	15 (62.5%)	13 (54.2%)	13 (54.2%)
2	32	15 (46.9%)	32 (100%)	14 (43.8%)	7 (21.9%)	8 (25.0%)
3	24	15 (62.5%)	14 (58.3%)	24 (100%)	14 (58.3%)	16 (66.7%)
4	18	13 (72.2%)	7 (38.9%)	14 (77.8%)	18 (100%)	14 (77.8%)
5	22	13 (59.1%)	8 (36.4%)	16 (72.7%)	14 (63.6%)	22 (100%)

**Table 6:** Number of species (and respective percentage values in parentheses) in the different categories (score values) of the three sub-indices DBS, TBS, and SBS. n/a = not assigned.

Score	DBS	TBS	SBS
0	n/a	29 (53.7%)	19 (35.2%)
1	12 (22.2%)	5 (9.3%)	9 (16.7%)
2	16 (29.6%)	17 (31.5%)	6 (11.1%)
4	25 (46.3%)	3 (5.6%)	20 (37.0%)
8	1 (1.9%)	n/a	n/a

**Table 7:** Number of sites and minimum, maximum, and median RABI for sites in each ecozone. In cases of the median RABI being split between two values, the arithmetic mean of the two values is given, indicated by *.

Ecozone	N Sites	Min RABI	Max RABI	Median RABI
1	6	11	47	15.5 *
2	21	5	63	33
3	8	14	33	18
4	2	16	29	22.5 *
5	14	5	30	14.5 *

## Data Availability

The original contributions presented in this study are included in the article. Further inquiries can be directed to the corresponding author.

## Abbreviations

The following abbreviations are used in this manuscript:

CR	Critically Endangered
DBI	Dragonfly Biotic Index
DBS	Distribution-based score
DD	Data-Deficient
EIA	Environmental Impact Assessment
EN	Endangered
EZ	Ecozone
IUCN	International Union for Conservation of Nature
LC	Least Concern
n/a	Not assigned
NP	National Park
NT	Near Threatened
RABI	Rwanda Anuran-based Biotic-Integrity Index
RDBI	Rwanda Dragonfly Biotic Index
SBS	Sensitivity-based score
SI	Species Index
TBS	Threat-based score
VU	Vulnerable

## Appendix A

**Table A1:** Sample sites in the respective ecozone. Overview of species number and RABI for each sample site.

**Ecozone**	**ID Locality**	**Locality**	**N Species**	**RABI**
1	11	Lake Kivu, Gisenyi–Marais	11	14
1	13	Kibuye–Marais	10	11
1	14	Cyangugu–Marais	12	16
1	16	Roadside Marais	7	15
1	17	Cyamudongo–Marais	11	30
1	28	Cyamudongo–Forest	9	47
2	3	Rukarara	7	20
2	10	Inselberg	4	6
2	12	Gishwati–Marais	8	19
2	21	Nyungwe–Source of Nile	6	37
2	22	Nyungwe–Uwasenkoko	10	63
2	23	Nyungwe–Nshili	7	39
2	24	Nyungwe–Rukuzi	5	39
2	25	Nyungwe–Busoro	6	30
2	26	Nyungwe–Kamiranzovu	9	57
2	27	Nyungwe–Karamba	6	43
2	29	Gishwati–Forest	10	63
2	30	Volcanoes–Lac Ngezi	8	33
2	31	Volcanoes–Gahinga Saddle	5	32
2	44	Gisakura–Marais	2	5
2	45	Kitabi	2	5
2	46	Nyungwe–Stream	4	38
2	47	Nyungwe–Waterfall trail	8	51
2	48	Volcanoes–Sandi	7	25
2	49	Volcanoes–Kabatwa	4	15
2	50	Nyungwe–Ndambarare Waterfall	7	53
2	51	Volcanoes–Malalo	6	27
3	1	Butare	17	35
3	2	Karama	10	14
3	4	Mwogo I	13	18
3	5	Mwogo II	11	16
3	6	Gitarama	14	20
3	8	Ruhengeri–Marais	11	18
3	9	Ruhengeri–southern River crossing	12	15
3	32	Rugesi–Marais	10	38
4	7	Kigali–Marais	12	16
4	15	Bugarama–Marais	13	29
5	18	Mugesera–Marais (Gashora)	15	26
5	19	Rusumo–Marais	7	8
5	20	Akagera River	14	19
5	33	Muyumbu I	5	14
5	34	Rwisirabo Hill Pond	4	5
5	35	Lake Shakani	8	10
5	36	Lake Birengero	7	9
5	37	Lake Mihindi	5	6
5	38	Bweya Ponds	8	25
5	39	Lake Rwanyakazinga	7	11
5	40	Kilala Plains I	5	15
5	41	Kilala Plains II	6	25
5	42	North of Kilala	10	30
5	43	Akagera Plains	10	16

**Table A2:** Overview of all species in relation to the number of sample sites where they were documented.

**Species**	**N Sites**
*Arthroleptis adolfifriederici*	8
*Arthroleptis nyungwensis*	4
*Arthroleptis schubotzi*	3
*Cardioglossa cyaneospila*	4
*Leptopelis bocagii*	8
*Leptopelis karissimbensis*	8
*Leptopelis kivuensis*	13
*Leptopelis* cf. *cynnamomeus*	1
*Sclerophrys berghei*	3
*Sclerophrys kisoloensis*	19
*Sclerophrys gutturalis*	19
*Hoplobatrachus occipitalis*	1
*Afrixalus orophilus*	1
*Afrixalus phantasma*	4
*Afrixalus quadrivittatus*	18
*Hylambates verrucosus*	1
*Hyperolius castaneus*	18
*Hyperolius cinnamomeoventris*	7
*Hyperolius discodactylus*	13
*Hyperolius frontalis*	1
*Hyperolius glandicolor*	7
*Hyperolius jackie*	2
*Hyperolius kivuensis*	27
*Hyperolius lateralis*	4
*Hyperolius parallelus*	4
*Hyperolius rwandae*	12
*Hyperolius viridiflavus*	19
*Kassina senegalensis*	23
*Phrynobatrachus acutirostris*	3
*Phrynobatrachus bequaerti*	4
*Phrynobatrachus graueri*	2
*Phrynobatrachus kakamikro*	16
*Phrynobatrachus natalensis*	20
*Phrynobatrachus* sp. *(natalensis group)*	1
*Phrynobatrachus parvulus*	3
*Phrynobatrachus scheffleri*	1
*Phrynobatrachus versicolor*	3
*Phrynobatrachus* sp.	1
*Xenopus victorianus*	13
*Xenopus wittei*	8
*Xenopus muelleri*	1
*Ptychadena anchietae*	21
*Ptychadena chrysogaster*	2
*Ptychadena guibei*	1
*Ptychadena nilotica*	24
*Ptychadena oxyrhynchus*	2
*Ptychadena porosissima*	10
*Ptychadena uzungwensis*	1
*Ptychadena* sp. A	3
*Ptychadena* sp. B	1
*Amietia desaegeri*	2
*Amietia nutti*	25
*Amietia ruwenzorica*	2
*Cacosternum plimptoni*	1

**Table A3:** Distribution of all documented anuran species within ecozones (number of ecozones in which the respective species occurs and percentage of unique species for each ecozone). Unique species are highlighted in grey.

	**Ecozone**					
**Species**	**1**	**2**	**3**	**4**	**5**	**N Ecozone/Species**
*Arthroleptis adolfifriederici*	x	x				2
*Arthroleptis nyungwensis*	x	x				2
*Arthroleptis schubotzi*	x	x				2
*Cardioglossa cyaneospila*		x				1
*Leptopelis bocagii*	x		x	x	x	4
*Leptopelis karissimbensis*		x				1
*Leptopelis kivuensis*	x	x	x			3
*Leptopelis* cf. *cynnamomeus*	x					1
*Sclerophrys berghei*		x				1
*Sclerophrys kisoloensis*	x	x	x			3
*Sclerophrys gutturalis*	x	x	x	x	x	5
*Hoplobatrachus occipitalis*				x		1
*Afrixalus orophilus*			x			1
*Afrixalus phantasma*		x				1
*Afrixalus quadrivittatus*	x		x	x	x	4
*Hylambates verrucosus*		x				1
*Hyperolius castaneus*	x	x				2
*Hyperolius cinnamomeoventris*			x		x	2
*Hyperolius discodactylus*		x				1
*Hyperolius frontalis*		x				1
*Hyperolius glandicolor*		x	x			2
*Hyperolius jackie*		x				1
*Hyperolius kivuensis*	x	x	x	x	x	5
*Hyperolius lateralis*		x	x		x	3
*Hyperolius parallelus*	x			x		2
*Hyperolius rwandae*	x		x	x	x	4
*Hyperolius viridiflavus*	x	x	x	x	x	5
*Kassina senegalensis*	x	x	x	x	x	5
*Phrynobatrachus acutirostris*		x				1
*Phrynobatrachus bequaerti*		x	x			2
*Phrynobatrachus graueri*		x				1
*Phrynobatrachus kakamikro*			x	x	x	3
*Phrynobatrachus natalensis*	x		x	x	x	4
*Phrynobatrachus* sp. (*natalensis* group)	x					1
*Phrynobatrachus parvulus*	x				x	2
*Phrynobatrachus scheffleri*					x	1
*Phrynobatrachus versicolor*	x	x				2
*Phrynobatrachus* sp.		x				1
*Xenopus victorianus*	x		x	x	x	4
*Xenopus wittei*	x	x	x			3
*Xenopus muelleri*				x		1
*Ptychadena anchietae*	x	x	x	x	x	5
*Ptychadena chrysogaster*		x	x			2
*Ptychadena guibei*				x		1
*Ptychadena nilotica*	x	x	x	x	x	5
*Ptychadena oxyrhynchus*					x	1
*Ptychadena porosissima*			x	x	x	3
*Ptychadena uzungwensis*			x			1
*Ptychadena* sp. A					x	1
*Ptychadena* sp. B					x	1
*Amietia desaegeri*		x				1
*Amietia nutti*	x	x	x	x	x	5
*Amietia ruwenzorica*		x				1
*Cacosternum plimptoni*					x	1
N species/Ecozone	24	32	24	18	22	
N unique species	2	13	2	3	5	
Unique species [%]	8.3	40.6	8.3	16.7	22.7	

**Table A4:** Overview of sub-indices and the resulting Species Index (SI) for all 54 documented anuran species.

**Species**	**DBS**	**TBS**	**SBS**	**SI**
*Arthroleptis adolfifriederici*	2	0	4	6
*Arthroleptis nyungwensis*	2	1	4	7
*Arthroleptis schubotzi*	2	0	1	3
*Cardioglossa cyaneospila*	4	2	4	10
*Leptopelis bocagii*	1	0	1	2
*Leptopelis karissimbensis*	4	1	2	7
*Leptopelis kivuensis*	2	0	2	4
*Leptopelis* cf. *cynnamomeus*	4	2	1	7
*Sclerophrys berghei*	4	2	4	10
*Sclerophrys kisoloensis*	2	0	0	2
*Sclerophrys gutturalis*	1	0	0	1
*Hoplobatrachus occipitalis*	4	0	0	4
*Afrixalus orophilus*	4	4	2	10
*Afrixalus phantasma*	4	1	4	9
*Afrixalus quadrivittatus*	1	0	0	1
*Hylambates verrucosus*	4	2	4	10
*Hyperolius castaneus*	2	0	2	4
*Hyperolius cinnamomeoventris*	2	0	1	3
*Hyperolius discodactylus*	4	0	4	8
*Hyperolius frontalis*	4	2	4	10
*Hyperolius glandicolor*	2	0	0	2
*Hyperolius jackie*	8	4	4	16
*Hyperolius kivuensis*	1	0	0	1
*Hyperolius lateralis*	2	2	2	6
*Hyperolius parallelus*	2	0	0	2
*Hyperolius rwandae*	1	0	1	2
*Hyperolius viridiflavus*	1	0	0	1
*Kassina senegalensis*	1	0	0	1
*Phrynobatrachus acutirostris*	4	2	4	10
*Phrynobatrachus bequaerti*	4	0	1	5
*Phrynobatrachus graueri*	4	1	4	9
*Phrynobatrachus kakamikro*	2	0	0	2
*Phrynobatrachus natalensis*	1	0	0	1
*Phrynobatrachus* sp*. (natalensis* group*)*	4	2	1	7
*Phrynobatrachus parvulus*	2	0	0	2
*Phrynobatrachus scheffleri*	4	2	4	10
*Phrynobatrachus versicolor*	2	1	4	7
*Phrynobatrachus* sp.	4	2	4	10
*Xenopus victorianus*	1	0	0	1
*Xenopus wittei*	2	0	0	2
*Xenopus muelleri*	4	0	0	4
*Ptychadena anchietae*	1	0	1	2
*Ptychadena chrysogaster*	2	4	2	8
*Ptychadena guibei*	4	2	0	6
*Ptychadena nilotica*	1	0	0	1
*Ptychadena oxyrhynchus*	4	0	4	8
*Ptychadena porosissima*	2	0	0	2
*Ptychadena uzungwensis*	4	2	1	7
*Ptychadena* sp. A	4	2	4	10
*Ptychadena* sp. B	4	2	4	10
*Amietia desaegeri*	4	2	4	10
*Amietia nutti*	1	0	0	1
*Amietia ruwenzorica*	4	2	4	10
*Cacosternum plimptoni*	4	2	4	10
